# A qualitative study to explore the lived experiences of people who are tilted ‘wubao’ within ‘three no’s’ category in government-funded care homes in China

**DOI:** 10.1186/s12877-025-06271-3

**Published:** 2025-08-23

**Authors:** Xiangping Fan, Rong Ding, Xiubin Zhang

**Affiliations:** 1 The Third People’s Hospital of Lanzhou, Lanzhou, 730030 China; 2 School of Public Health, Imperial College London, Sir Michael Uren Building, Wood Lane 86, White City, London, W12 7RQ England; 3 Present Address: Medical School, Lancaster University, Bailring, Lancaster, LA1 4YW England, UK; 4 School of Nursing, Lanzhou University, Lanzhou, 730030 China

**Keywords:** China, Government-funded care home, Older people, Qualitative study

## Abstract

**Background:**

There are limited studies explored the lived experience of older people without children who titled ‘Wubao’ in China. This study aims to understand the lived experience of the older people who titled ‘Wubao’ and fit in the ‘Three No’s’ category in government-funded care homes in China.

**Methods:**

The phenomenological qualitative approach was used for this study. Thematic analysis was used for data analysis.

**Findings:**

Three main themes and six subthemes were identified. Theme 1: It has significantly changed basic life needs (availability of physical life resources; availability of basic medical care). Theme 2: Lack of personal-centered care services (lack of specialized dietary needs; lack of care equipment to support personal needs). Theme 3: Social attitude of living in government funded care homes (losing social connections; perceiving social stigma).

**Conclusions:**

The findings highlighted that the government-funded care home is important to support and meet the basic physical needs for this group population. However, the personalized needs seem to be underestimated in this type of care home. It suggests that this type of care home should pay more attention to the older people’s emotional needs, and the government should consider how to ensure social inclusivity for them.

**Supplementary Information:**

The online version contains supplementary material available at 10.1186/s12877-025-06271-3.

## Introduction

China has entered the ageing society, it was recorded that there were 249 million people over 60 years old and 167 million people over 65 years old, accounting for 17.90% and 11.90% of the total national population in 2018 respectively [1, 2]. With the ageing population continuously increasing, caring for the older people has become an inevitable social issue that has become a focus of society, government and academia. Currently, China has no national pension system for older residents. A working pension is the main income for retirees, however, this only applies to people who have had a formal job before they were retried [3]. People without a long-term, full time job are less likely to have a pension, especially rural residents who work on their own land [4]. According to one survey, it was estimated that two thirds of the older population lived in rural areas where many state welfare provisions are limited [5], and most of them lacked retirement pension support. Therefore, most older people who live in the rural areas are supported by their children [6, 7]. Furthermore, the traditional cultural norm and the law of the Rights and Interests of the Older Persons in China also requires adult children to take responsibility of caring for their older parents. This kind of care arrangement means that older people would be financially and physically dependent on their children ([8–10].

Among the population of older people without a pension, there are some older people without family or children to take care of them. If they have no children, no physical ability to earn a living cost at age 60 and no income, then they are categorized as the ‘Three No’s’ [11, 12], they would then be eligiable to access the Chinese government’s special funded care home for their aged care as the traditional family-based care would not posible for them. This support care is called the ‘Five Guarantees’ pragram [12–14], which assures this population group would be provided with, at least, food, clothing, housing, medical care, and burial expenses. These older adults are usually called ‘Wubao’ (literally translates as five guarantees) [11, 13, 15–17]. This specific aged care system is an important care arrangement in supporting older people who are childless or without family [18, 19]. In 2014, China had 5.29 million ‘Wubao’ recipients in rural areas [11].

The living experience of older people in nursing homes have already been explored in many studies in different countries [20–26]. However, until now, there are no studies exploring the living experience of this older, ‘Wubao’ population, who live in Chinese government-funded care homes. Therefore, this study explored the insight of this special group in the context of governmental funded care homes in China.

## Methods

### Study design

A phenomenological qualitative approach was used to explore the lived experiences of participants. Phenomenology focuses on understanding how individuals perceive and make sense of their life experiences. It aims to capture the essence of these experience by emphasizing interpersonal processes such as motivation, stress and perception [24]. In particular, phenomenology is well-suited to examining how individuals interpret major life events and the meanings they ascribe to their lived experiences. This method allowed us to deeply explore the subjective realities of older individuals residing in government-funded care homes in China.

### Study aim

The aim of this study was to understand the lived experiences of these older people who without children and fall in ‘Three NO’s’ category, living in government-funded care homes in China, to explore the views and needs of the older people and find out what can further promote and improve the quality of their life and care. This investigation is particularly important as this vulnerable population often faces social and medical marginalization.

### Research setting and participant recruitment

A purposive sampling approach was used to focus on the specific population group relevant to our research topic. Participants were recruited from two government-funded care homes in China. One care home had 100 ‘Wubao’ older residents, 15 staff including the management team, carers, catering staff, cleaners, but no qualified healthcare professionals. Another care home had 50 beds, 45 of which were occupied, all by ‘Wubao’ residents. There were seven staff on duty, which is similar with the first care home. Both care homes only provide basic physical care such as washing, cooking, dressing services to the older people. They did not provide medical support. The older people would be transferred to a local hospital if they needed medical care. Moreover, none of the care staff received pre-work training as caregivers.

Participants had to meet the following eligibility criteria: (1) aged over 60 (the retirement age in China); (2) classified under the ‘Three No’s’; (3) residing in a government-funded care home in China for over two years; (4) capable and willing to participate in the study.

The care home management teams helped to deliver the study information sheet and consent forms to the older people in these two care homes. Then an initial meeting was held by the first author at each care home to explain the study and answer any questions. Twenty people expressed interest and sixteen agreed to participate and signed the consent forms (10 from one care home and 6 from the other). Four declined to participate, citing a lack of interest or unwillingness to give consent.

### Data collection

Semi-structured, in-depth individual interviews were used for data collection. An interview guidance with open questions was developed in advance to allow for the exploration of topics that are important to answering the research question. The interview questions focused on exploring the participants’ daily activities in the government-funded care home, their perspectives on living there, and their feelings about being ‘Wubao’ residents. For example, we asked: ‘Can you describe a day in the care home?’ and ‘Can you tell me about your experiences of living in the government-funded care home? Face to face interviews were conducted by two team members, one facilitated the interviews and another one acted as a notetaker, documenting observations and reflections. All interviews were audio recorded and each lasted approximately 45–60 min. Data collection and transcription was conducted simultaneously in order to create more opportunities for revising or prompting new questions. After 16 interviews were conducted, no new information was presented, and data saturation was reached. Each participant was interviewed once as we find that sufficient data had been gathered to support the key themes and topics in detail. All interviews were transcribed verbatim.

All researchers involved in the study were trained in qualitative research methods. One holds a PhD in Nursing, another has a Master’s degree in Psychology and is pursuing a PhD, and the third is a Registered Nurse. The researchers were mindful of their positionality and adhered to ethical guidelines to ensure that their personal biases did not influence data collection or analysis. No prior relationships were established, and none of the researchers knew the participants before the interviews. The COREQ (Consolidated Criteria for Reporting Qualitative Research) checklist has been attached as an appendix to provide further details regarding the research team, study design, analysis, and findings (see appendix 1).

#### Data analysis

Thematic analysis was used for data analysis (Braun & Clarke, 2006). Firstly, the researchers familiarized themselves with the original database. Then, line by line, initial comments and codes were made to form the initial codes by an experienced researcher. Then the initial codes were identified and grouped into initial emerging themes. At this stage, two researchers checked the reliability by going back to the original data and matching the initial codes with each coded transcription. After all the transcriptions were analysed, all the coded data was grouped to identify the patterns and connections by using a thematical map. Finally, a master table was developed to present and compare the emerging themes across all the transcripts, identifying and consolidating the final themes and sub-themes.

To ensure rigor, the findings were sent back to participants for member checking, allowing them to confirm that the themes accurately represented their experiences. The research team also frequently met and discussed any issue raised during the analysis process and ensure to reach an insistent outcome. NVivo12 software was used to manage the data and assist in the analysis.

## Results

The 16 participants ranged in age from between 62 and 85 years old and their duration of living in the two care homes ranged from 2 to 10 years, 13 out of 16 were illiterate. 7 females and 9 males.

Three main themes and subsequently six subthemes were categorised and identified: Theme 1: It has significantly changed basic life needs (Availability of physical life resources; Availability of basic medical care). Theme 2: Lack of personal-centered care services (Lack of specialized dietary needs; Lack of care equipment to support personal needs). Theme 3: Social attitude of living in government funded care homes (Losing social connections; Perceiving social stigma). These themes disclose a series of care home life stories, care services and everyday activities of living in these specific care homes.

### Theme 1: It has significantly changed basic life needs

This theme reflected how the care arrangement has significantly changed the older people’ lives, what their perceived experiences are, their expectations and what the meaning of life is for them after living in the care home.

### Availability of physical life resources

In the study, most participants expressed their satisfaction with their physical care of living in the government-funded care homes. It has significantly changed their basic life needs. For example, participant 5 made it clear that the system provided them with essential needs, such as food, clothes and shelter. Moreover, the supplements which were provided for him also kept him satisfied.

*“Everything is supportive to us. Thank President Xi for his good policy*,* it’s hard to imagine my life without it…*,* I used to live in my humble house*,* now there are two people per room which is equipped with electric heating. There are public dining rooms and other leisure spaces…’’ (P5)*.

The following extract also showed similar responses from participant 16. Living in the government-funded care home has given the participant a sense of happiness due to the reliable living conditions.

*“In the past*,* I lived with my older father and older brother. I had an old injury to made me difficult to move around. No one cared about me after they passed away*,* now I am happier because the government manages our life*,* food*,* and clothes.” (P16)*.

Similarly, participant 9 expressed that their basic daily care needs have been met at the care home, care staff have arranged to take charge of their daily activities, such as providing three meals every day and washing clothes. Therefore, he has no concerns about his life now, which reflect his satisfaction and happiness because of the better living conditions.

*“I didn’t have meals on time at home…*,* now my life is better than before*,* I have three meals on time every day*,* and I do not need to worry about anything in here.” (P9)*.

### Avaiablility of basic medical care

Most participants expressed that the “five-guarantees” system also provides medical care, for example, a particular quote from participant 8 showed that how they received medical care while living in the care home.

*“All medical expenses are paid by the government*,* sometimes doctors come to the care home for free consultations.” (P8)*.

Participant 10 suffered from rheumatoid arthritis, cervical osteo hyperplasia and gout, which led him to be semi-disabled and he lost the skills to take care of himself.

*“In the past*,* my leg is painful*,* I couldn’t take a pail of water into the room and had no meals on time…*,* it was not convenient for me to buy things*,* also I didn’t have enough money. Now the government takes care of us*,* when my leg gave me a lot of pain*,* doctors from the town hospital came to check and treated it for me*,* my leg is getting better now…. Without this policy*,* people like me would have starved to death.” (P10)*.

Poor health conditions stopped participant 10 from getting out of his room in the past, this affected his self-care ability in his daily life before he was administrated to the care home. Along with a lack of financial resources, his living conditions were very bad. Fortunately, the government funded care home had saved him. When he said, ‘Without this policy, people like me would have starved to death’, it illustrated that life has changed dramatically for him after living in the care home. Not only does he get meals on time, but the quality of his life has also been greatly improved, what is also important is that it has given him hope to live, which has changed the meaning of life for him.

However, a sense of being unsupported by health professionals was also evident for all participants. For example, participant 10 with chronic diseases said that sometimes it’s hard for the government to continuously provide them with some necessary drugs for their disease. He is disappointed that continuous health care was not provided after his body was checked.

*“After they finished*,* no medicine review service for us (after was sent to us…If some necessary drugs for diseases are given to us after being checked*,* then that would be better.” (P3)*.

A similar situation was revealed by another participant 11, who felt that medication was not provided on time.

*“I have lung disease*,* I feel chest tightness. There is no cephalosporin here*,* I went out and bought medicine on my own*,* it cost me lots of money.” (Participant 11)*.

### Theme 2 Lack of personal-centered care services

This theme mainly reflected the participants’ demand for person-centred care services and psychological needs.

### Lack of specializes dietary needs

This subtheme highlights that there lack of specializes dietary needs for individuals in this type of care home. For example, participant 2 talked about the care home’s failure to meet his personal dietary preferences, which lead him to feel frustrated and disappointed.

*“My teeth are too old to chew food properly*,* so I could not digest the hard food*,* it is a shame.” (P2)*.

Participant 13 has perceived the same experience related to food provided by the care home.

*“I suffer from heart disease and high blood pressure; I can’t eat meat or salty food. When the food is salty*,* I will soak it in boiling water before eating.” (P13)*.

### Lack care equipment to support personal needs

In the care homes, there are some entertainment facilities, but most are underutilized with the room doors being closed due to fear of accidents. Moreover, some facilities are not suitable for older people. For example, participant 1 says:

*“There is a room where entertaining facilities are available*,* such as mahjong*,* table tennis*,* and other sports equipment such as a bicycle. But the room is locked*,* and facilities are not made full use of*,* maybe they (care staff) are afraid of older people getting injured.” (P4)*.

It not only indicates that some care services may not effectively benefit older people in such care homes, but also reflects the unapplicable caring approach taken in the care homes.

In addition, most participants are illiterate or semi-literate, their recreational activities are limited, the care home rarely organizes any social events for them. Thus, the older people can only watch TV in their rooms or sit in their room for the whole day. At present, there are few psychological/emotional support activities organized, so psychological support and social interaction is particularly low, which can also affect mental health through loneliness or low self-esteem. For example, participant 12 said:

*“We don’t have any entertainment activities*,* I am illiterate*,* so I cannot read*,* I usually watch the TV or sit in one place for a whole day” (P12)*.

Even though care homes provide convenient basic care services to older people, the care services provided follow a routine care approach, which lacks consideration of personal preferences. In consequence, the older residents felt that they were overlooked and not respected by caregiving staff who never asked them about their preferences. For example, Ha says:

*“They never ask me what I want*,* as for me*,* sometimes I would like to have my own time. if I stay at my own home*,* I could freely to arrange my time and will be more happy” (Participant 2).*

It shows that management and care services in the care home did not make him feel that he had explicit authority and that it did not provide the freedom he would have had at home. This caused Ha to feel a sense of disrespect and dissatisfaction, and also reflects that person-centred care has been ignored in this care home.

Theme 3: Social attitude of living in government funded care homes

### Theme 3: Social attitude of living in government funded care homes

The theme demonstrated that the older person perceived not only limited social support from outside of the care homes, but that ‘negative attitudes’ also emerged due to the lack of social connections and interactions.

### Losing social connections

As the participants have no family, so there are very few family visitors visiting the care home, so once the older person has been administrated to a government funded care home, it might mean that the person is cut off from the outside world. In China, the traditional culture is to get together with family members during festivals. Participants feel a sense of loneliness during the traditional festivals. For instance, Ma says:

*“Hardly anybody visit me*,* on festive*,* such as spring festival…*,* if I see other people get together with their visitors to celebrate*,* I feel lonely.” (P10)*.

The participant perceived a feel of loneliness perhaps reflecting his psychological need for support from the care home staff. It also indicates the importance of providing emotional support for older people who have less family or relatives’ support as they have fewer social connections and interactions than other older people who have a family.

In the study, some participants indicated that there are hardly any organizations, companies or individuals that pay attention on them. There are also very few voluntary or social networks involved in the support of the care homes.

*“Some organizations visit us occasionally*,* such as*,* students from a local school organize activities for us*,* they sang and danced for us. In last summer*,* the police brought fruit and visited us.” (P3).*

Even though there were a few donations or support from some organizations, these kinds of donations are more of a staged ‘show’, their main purposes are to improve their organizations or companies’ reputation, so it is more focused on the social influence of the donators’ rather than real social support for the older residents.

Similar common reactions were described by participant Chen. Older people have perceived a negative view of social support. The following statement shows how older people with no children feel neglected by society.

*“Although we are looking forward to and pleased with their arrival*,* few people visit us.” (P7)*.

The statement from Chen has shown how the older person is eager to gain attention from the outside world. More importantly, it also reveals the lack of social attention and support for this population group.

### Perceived social stigma

In the study, social attitude toward this type of care home and older people without children is another important issue. The following quotes Feng’s, which describes his feeling of being ashamed and disrespected. In his view, the phrase ‘people who lived in the care homes look like animals’ disclosed the social attitudes towards them and demonstrates how society views these older people as useless. This bias and discrimination not only caused frustration and loneliness, but also an aggravated sense of stigma due to childlessness. What’s more, ‘older people living in care homes are just like animals in cages’ also indicates that they lack interaction with the outside world and freedom. This had affected their dignity, resulting in the older people’s isolation, hopelessness and desperation.

*“Well*,* I feel they show no respect to us*,* I once heard from people outside who said that we lived in care home look like animals*,* being feed but no social connections and contributions.” (P1)*.

Attitudes from people outside the care home have great influences on their wellbeing and behaviours. Participant 1 has not only perceived disrespect from others, but also experienced social discrimination from the society by living in the government care home. This social attitude and stigma toward people who do not have children has made participant 1 feel hurt and insecure.

Participant 16 described his relatives’ attitudes after he has been administrated to the care home. He raised the issue that his relatives and friends have never visited him after he came to the care home which made him unhappy and felt that he was being overlooked.

*“My relatives and friends have not been to visit me since I came to the care home*,* it makes me sad.” (P15)*.

This indicates not only the lack of social support and connection but may also lead the participant to suffer from loneliness and stress.

## Discussion

This is the first study to explore the lived experience of older people without children who titled ‘Wubao’ live in government funded care homes in China. It provides a new perspective from the older people’s viewpoints to reveal what their real life looks like in this specific care setting. In the study, most older residents who live in the government-funded care home are satisfied with their basic life demands, such as food and clothing. It has significantly changed the life of some older residents who are without children to support their care. This type of care approach may have potential to be implemented in other countries where older care services are developing, especially for the older population at the deprived areas.

However, special, or personalized needs for some older people were unmet, such as dietary needs for some participants due to their dental problems. Some older residents complained about this situation. Since personal centred care has been on the healthcare agenda, it has shown to advance concordance between care provider and patient in terms of treatment plans, improved health outcomes and increased patient satisfaction. One study indicates that personal cantered care or tailored activities could improve residents’ wellbeing [25]. Thus, the care service should also consider older people’s preferences, and their special needs should be fully respected and satisfied. Furthermore, in this study, most older residents were illiterate, reading books and newspapers was beyond their ability. Additionally, there are few recreational activities that can take place in the care homes. These factors may have led the older people to experience loneliness and dissatisfaction. It indicates that the care home needs to consider the spiritual needs of the older people and to improve the overall quality of support. Respecting the needs and preferences of individuals are important components in personal-centred care, this should be considered and made available in these types of care services.

Another issue is that care staff in both of the care homes were unqualified rural women with a limited educational level, as in most other institutions in China [2, 11]. This reflects the lack of qualified professional care in this type of care home which has significantly affected the quality of the care provided. This also may be the reason the care homes used a routine care approach to provide daily care for the older people, rather than providing person-centred care. This issue could be solved by training or recruiting qualified care professionals and establishing a holistic care approach in the care homes. Previous studies also show that more than 60% of the old age care institutions in China have no professional nursing staff, and more than 50% of institutions have no doctors [2, 26]. In response to the inadequately supplied medical care, there is a need to encourage health professionals to work in the care institutions [27]. Cooperation mechanisms between medical institutions and older care institutions may be worth trying. For example, medical institutions could provide medical care training to care staff in care institutions, and regularly conduct basic diagnosis and treatment services such as detection of common diseases, the management of chronic and geriatric diseases, as well as implement health education in the care homes [2]. This way, we can not only meet the basic life care needs for the older people but also guarantee medical care for them [28, 29], it will also effectively solve the shortcomings of the medical service in the current care homes, and improve the overall level of care service.

In UK settings, the contrast between medicalised care and personalised care is well established, with the latter emphasizing a holistic approach that goes beyond diagnosis to consider an individual’s broader needs and preferences [30, 31]. While personalised care empowers individuals, the integration of medicalised care might work differently in non-Western cultures. In China, where the healthcare and aged care systems are still developing, aligning medical care with cultural values adds complexity [32]. As China reforms its system, ensuring personalised care is culturally appropriate presents unique challenges that require further research and attention from policymakers.

From the theme “Social attitude of living in government funded care homes”, we found that the public is less aware about the involvements and concerns of the older people’s life in such care homes. Even though the ‘Five Guarantees system’, assured at least minimal coverage with food, clothing, housing, medical care, and burial expenses, it brought social stigma to the person who identified as a ‘Wubao’. In Chinese culture, having no descendant is one of ‘three unfilial acts’ [33]. In combination, childlessness and poverty create a heavily stigmatized cultural identity, so this group population are more likely to be treated as ‘losers’ or ‘failures’, and considered as a disgraceful and pathetic group in the society [34]. Previous studies also reported that biases and discrimination from people outside the care home caused older people to feel frustrated, lonely and that they have lost their dignity due to others’ negative attitudes [35, 36]. This suggests that both care homes and policy makers should strengthen public awareness and social support for this group of older people. For example, advocating the public to volunteer in care homes by providing some physical and emotional support, as well as to promote the lay population to understand the old people’s life. This action can protect older people from discrimination and isolation. In addition, the traditional Chinese norm of ‘more children more blessings’ and the traditional family-based care culture has made some childless older people more likely to be treated as an inferior group in society. This cultural perception has obviously increased the disconnection and social stigma towards older people who were childless [37]. It indicates the indispensable need for a change in social attitudes toward the population group. Healthcare and long-term care services programs should be integrated into the care homes or replace the ‘Five Guarantees’ program to reduce cultural factors causing social stigmatization.

### Study Limitations and Strengthens

The study was conducted in one care home which was funded by the Chinese government, it specialises in caring for older people without children. The care home type, management, economy and geography are unique. The results of this study perhaps can’t be generalised to other types of care homes. The thematic analysis of this qualitative study we employed may not represent a novel methodological advancement, but we chose it for its suitability in analysing the experiences of the ‘Wubao’ population within the specific social context of our study. Its strength lies in the novel application of this method to an underexplored population, given the limited existing research on this topic. The study serves as a ‘window’ into the living experience of the childless older population who are cared for at government-funded care homes in China. It raises awareness in considering the ethical issues and cultural factors when designing and implementing a care approach. This is the first study to explore the lived experiences of the service users in a unique care setting through using a qualitative approach.

## Conclusion

The study gives a glimpse into the living experience of childless older people who live in government funded care homes in China. These findings indicated that the government funded care homes under the ‘Five Guarantee’ program has significantly supported the basic life needs for this population group who are categorized as the ‘Three No’s’. However, to a certain degree, living in this specially funded care home by the government has also become a factor of social disconnection and isolation for this population group. The study suggests that it is important that not only the care homes pay attention to meet the needs and mental state of this group, but policy makers and wider society should also pay attention to them. It also suggests that more research is needed to explore more ways to support these older people.

## Supplementary Information

Supplementary Material 1.

## Data Availability

The full dataset and data analysis code following receipt of ethics approval may be available from the corresponding author.
